# Anæsthetic

**Published:** 1877-09

**Authors:** L. G. Noel


### ANÆSTHETICS.

BY L. G. NOEL. D. D. S.

*Read before the Tennessee Dental Association, June 27, 1877.*

So much has been written, and so many theories advanced upon the physiological action of anæsthetics, that the student of this branch of surgery is confused in the outset, dazed and overwhelmed with the mass of learned ignorance with which the subject is entangled.

Theorists in this branch of therapeutics have been divided into (1) those who believe in the comparative innocency of these agents, and (2) those who hold them all as hurtful to health, if not dangerous to life. The hypothesis of the first class, has been of a kind so specious as to cover up many facts of an alarming character, while those of the latter, have enshrouded the subject in a gloom of such cimmerian blackness, as to appall the timid, and deprive suffering humanity, in many instances, of that which would have been a harmless blessing.

When we take up the long list of agents that have been used as anæsthetics, we find we may divide them into two classes; 1st, anæsthetics rich in oxygen; 2nd, those devoid of oxygen, or containing that element in very small proportions. Of the first class may be mentioned, nitrous oxide, ether, pure oxygen, atmospheric air, and the alcoholic stimulants. Of the latter, chloroform, bi-chloride of methelene, methylic ether, compounds of amyl, and the tetra-chloride of carbon.

Now the theory which of all others strikes us, as most probable in regard to the action of the first class is this:

Being rich in oxygen, that agent enters into the blood in quantity, far transcending the process of normal respiration, and by its union with the tissues, carbonic acid is formed in such quantity, as to render it impossible for the lungs to eliminate it from the economy. The arterial blood thus poisoned is sent to the brain, and its narcotic effects are impressed upon that organ. We believe the same to be true of all oxygenated anæsthetics, nor do we think this a more alarming view to take of the subject, than that these agents act as narcotic *per se.*

No one thinks of being seriously alarmed for the life of the drunkard, who is found peacefully sleeping upon the sidewalk; yet he is in a state of anæsthesia, almost as profound as that produced by ether or nitrous oxide. The oxygen of the alcohol has set to work upon his tissues, and his brain is stupified by carbonic acid. In the anæsthesia of alcohol, the narcosis is much less rapid, and for that reason probably less profound.

To this view of the action of the oxygenated anæsthetics, it may occur to the superficial student, that pure oxygen gas should then produce the most profound narcosis, and be the most valuable of these agents. We must remember, however, that gases are most active—most eager to seek new affinities, when in a nascent state. In other words a recomposition is much more readily affected from the heat of a decomposition. The breaking up of the molecule of ether, or alcohol as the case may be, leaves its oxygen atoms nascent, and all eager to form new alliances. Then beside this nascent activity, we have the catalytic action of other elements present, to assist in the process. No one who has had any considerable experience in the administration of nitrous oxide and ether, and who has observed the purple blush of the skin, the rapid laboring of the lungs and heart, can, it seems to us, doubt the correctness of this explanation.

The non-oxygenated compounds seem to be much more direct in their action, impressing themselves at once upon the brain and nerve centers, without the generation in the blood of a secondary product, as the active agent. They seem to have an action *suo marte* upon the great centers of life at the base of the brain, and when these are enfeebled or diseased, they yield readily, become overpowered by the drug, and the processes of life cease.

It has been held by some, that anæsthetics all act primarily as stimulants to the brain, promoting an unwonted flow of blood to the encephalon, which establishes a hyperæsthesia, only passing into a condition of anæsthesia, when the blood vessels are so engorged with blood as to produce pressure upon the nerve centers, whereby their action is interrupted, and the cognizance of external injury is suspended. The condition of cerebral excitement in the earlier stages of anæsthesia, followed by drowziness and unconsciousness, would render this view plausible, nor is it inconsistent with the theory of universal narcosis, since it is well known that such is the *modus operandi* of opiates. The statistics that have been gathered upon this subject, prove conclusively that the non-oxygenated compounds are the most dangerous, and that they should seldom be employed as anæsthetics. Statistics collected by Dr. Chapman, show that during 1858 and the first six months of 1859, there were fourteen cases of death from anæsthetics in all parts of the world. He showed that up to 1867 anæsthetics had been administered to 1,200,000 persons including a period of ten years, and that during this time the deaths were one in 16,216. From statistics collected from fourteen hospitals, in London and Liverpool in 1867, it appears that chloroform was administered in 83,059 cases, of which 24, or one in 3,461, proved fatal. Professor Andrews, of Chicago, in 1870, collected from different American and European hospitals, the statistics of 117,078 cases, in which chloroform had been administered with 43 deaths, affording thus, a ratio of mortality of one in 2,723. Of 92,815 cases obtained in the same way of etherization, only four perished, or one in 23,204. A mixture of chloroform and ether was employed in 11,176 cases, of which only two died, the mortality being as one to 5,588. Dr. F. R. Thomas, of Philadelphia, claimed that he had up to September 1871 administered nitrous oxide gas in 22,950 cases for the extraction of teeth, without a single fatal example. He had administered the agent with perfect impunity, in disease of the heart and bronchial tubes, and in one instance with safety in advanced stage of phthisis.

(Gross) Dr. Bartholow, of Cincinnati in his excellent work on Materia Medica, has advanced the idea that a condition of incomplete anæsthesia is always a condition of danger. He says, "Numerous accidents have occurred from the use of anæsthetics for trivial operations, notably for extraction of teeth. In such instances the heart enfeebled by chloroform narcosis, is suddenly paralyzed by reflex action proceeding from the peripheral injury. The district of tissues supplied by the fifth nerve, is an especially dangerous region owing doubtless to the intimate connection of the nucleus of the fifth with the nucleus of the pneumogastric. By far the greater number of fatal cases have resulted from the neglect of this rule. It is never safe to proceed in a surgical operation with anæsthetics, unless complete anæsthesia has been produced.

Now a few words as to the administration of anæsthetics. The following rules should be observed in their exhibition: 1st examine carefully your patient before consenting to proceed with the drug, and satisfy yourself that the heart, lungs and brain are free from organic disease. Dentists are too careless about this matter of preparatory examination, and it is unfortunately true that too few of them have acquainted themselves with the physical diagnosis of the diseases to which these organs are liable.

2nd. Do not, except in cases of great emergency, administer these drugs to persons suffering from an emphysematous condition of the lungs, enlarged tonsils, acute laryngitis or diphtheritic swellings of the throat. We have in many instances witnessed the safe administration of anæsthetics, in cases of advanced phthisis, and believe it better often times to risk the drug than the shock attending an operation without it. The same remark is true of advanced pregnancy, in which condition it often becomes necessary to extract aching teeth. Having satisfied yourself as to the soundness of these organs, it is important to come to a definite understanding with your patient. Let him know that he is to submit himself fully to you, and do just as you direct him, that you can consent to operate only upon these conditions, that he pledge himself to submit, and offer no resistance, even though he should be conscious at the time you commence to operate.

For fluid anæsthetics, no inhaling is necessary other than a cone, made of a starched napkin, or a sheet of letter paper, with a bit of sponge placed in its apex, upon which to pour the anæsthetic.

Gags and corks for keeping the jaws apart are dangerous appliances, and we would rather take the risk of having to pry the jaws open, than expose the patient to the peril of getting these things into the trachea. No surgeon should, except in great emergency, undertake to administer an anæsthetic without the presence of an intelligent assistant. The surgeon cannot divide his attention between the effects of the anæsthetic, and the operation.

